# The physician's role in mitigating the climate crisis

**DOI:** 10.1016/j.fhj.2024.100173

**Published:** 2024-09-06

**Authors:** Thomas Hughes

**Affiliations:** Liverpool University Hospitals NHS Foundation Trust, United Kingdom

**Keywords:** Climate change, Global warming, Net-zero, Healthcare emissions

## Abstract

Climate change is a growing concern and healthcare is simultaneously being burdened with worsening climate-related health conditions, while significantly contributing to emissions and temperature rises. Studies have documented the effects of emissions on increasing numbers of early deaths, while rising temperatures and altered rainfall patterns lead to a change in vector migratory patterns alongside broader physical and mental health impacts. These changes only exacerbate the current global health inequities. The role of doctors in reducing the burden of the climate emergency can not be understated, including both individual and systemic changes. Preventative medicine can promote an active lifestyle and reduce fossil fuel consumption, while patient education and empowerment on reducing red meat consumption can improve cardiovascular health and reduce emissions. Low carbon solutions can be achieved via smarter prescribing and lean pathways can increase efficiency. It is in everyone's interests for physicians to adjust their practice to improve sustainability.

## Introduction

The phrase ‘climate change’ evokes images of distressed wildlife among acres of deforested land accompanied by a reprimanding voiceover from David Attenborough. However, the impact can be felt closer to home as I, again, talk a worried mother through the correct use of a spacer for her child’s new inhaler. Mere streets away from the practice I am working at, signs in Liverpool’s city centre warn that this is an area of high air pollution, a concerning indicator that the burden of chronic respiratory illnesses like asthma will only continue to grow.

The threat of the climate crisis has long been ignored over problems seen and felt on a day-to-day basis. Now the effects are becoming apparent and the sequelae will begin to affect every body system. Climate change is likely to have a greater impact, with a more long-lasting effect on both health and healthcare, than many of the worst pandemics humanity has experienced. It is in both the patient’s and the physician’s best interests to help mitigate it.

## The impact of the climate crisis

The effects of climate change stretch beyond heatstroke and hypothermia. Long-term exposure to air pollution has been linked to over 30,000 premature deaths in the UK per year, not accounting for the effect of damp homes on worsening chronic respiratory conditions.^1^ The change in temperature and rainfall patterns also increases the spectrum of diseases that populations are exposed to, while increasing exposure to high temperatures and heatwaves poses direct health risks, across several physical and mental health outcomes. The migratory patterns and habitats of animals and insects are dictated in part by the weather, changes in which can increase the number of people at risk of zoonotic and vector-driven infections such as malaria, tick-borne disease, and viral infections more broadly. It is estimated that an additional 4.7 billion people could be affected by mosquito-transmitted diseases by the end of the century if temperature rises are not further controlled.^2^

On a similar note, increased rainfall and rising sea levels escalate flooding risks, opening up a myriad of health problems. This reduces access to potable water, leading to dysentery and numerous water-borne infections. An understated impact of flooding is the effect on mental health, an area of healthcare that is increasingly at risk of becoming overwhelmed in Merseyside. UK studies have demonstrated the increase in risk of depression and anxiety that is associated with flooding due to the risk of displacement from one’s home and loss of belongings.^3^ The effects of flooding on a community can be compounded by flooded healthcare facilities, hindering the delivery of high-quality care and reducing the ability to admit patients at a time where need will be greatest.

Crop growth is particularly sensitive to weather changes; this is most pronounced in increasingly arid tropical areas. The UK Health Alliance on Climate Change estimates that over 80% of cultivated land in the UK is used for livestock, with most UK produce being imported, meaning that impacts on crop yields abroad will impact UK food security.^4^ Switching to a plant-based diet would reduce the demand for animal products, thus allowing more land to be used in growing crops and improve our food security. Without these changes, approximately 5% of the UK population are currently estimated to be at risk of undernutrition and this figure may go up as prices rise due to crop yields abroad falling.^5^ Imported crops are cheaper than home-grown foods and so this reduction in yields is likely to disproportionately affect the most disadvantaged sector of the population. The rising cost of living is a common fear shared by many of my patients seeking time off work for stress, and is evidence of the compounding health problems that disproportionately affect members of a lower socioeconomic status.

From respiratory illnesses to deteriorating mental health, the climate crisis is manifesting as a healthcare crisis, thus highlighting the importance and benefit to physicians in battling it.

## The role of the physician

Sustainability is increasingly expected of physicians, including from the General Medical Council, who have adapted their Good Medical Practice document to advocate ‘choosing sustainable solutions’ and ‘making good use of resources’.^6^ However, they have also come under scrutiny following the suspension of a doctor engaging in a peaceful climate protest.^7^ Notably, the Green physician toolkit from the Royal College of Physicians and the Centre for Sustainable Healthcare offer more actionable advice and training opportunities, highlighting the roles that doctors play in implementing both systemic and individual changes to tackle the climate crisis.

The NHS is committing to net zero emissions among controlled emissions by 2040. The plan aims to address emissions in all aspects of care delivery including reducing fossil fuel emissions and electricity consumption, as well as anaesthetic gas emissions. The changes can already be seen with desflurane, which is currently being decommissioned due to its high environmental impact compared to alternatives like sevoflurane.^8^ It may seem that the individual physician has little role to play in implementing systemic green change in the NHS. However, numerous anaesthetist colleagues who I speak to about the climate crisis have shared that they are switching to total intravenous anaesthesia in lieu of volatile gases, and this is now being recommended nationwide where possible in the Green Surgery report.^8^ Furthermore, physicians are considered as among the most trusted professions and have a loud voice in society, evidenced by the successful campaign leadership by doctors for the implementation of the Ultra Low Emission Zone to Greater London; this has resulted in a drastic reduction in air pollution in the area.^9^ Through activism, physicians have implemented systemic changes in a multitude of issues for the benefit of society.^10^ We are well placed to advocate for systemic changes to prevent worsening of the climate crisis, from campaigning for emission reductions to managing damp homes.

Within the workplace, facilitating more efficient services is a key pathway in managing climate change and is well within a doctor’s remit; my colleagues and I have recently been pushing for sustainability teaching to be incorporated into our foundation teaching programme in order to identify and act on inefficiencies in the workplace. From an outpatient perspective, patients are brought into clinics every day by taxis and patient transport ambulances to be run through test results or check their progress. This can be streamlined by integrating overlapping services, such as renal and diabetic clinics, and opting for remote clinics when possible. During the pandemic, clinics were nearly exclusively remote and inefficiencies came to light and were (mostly) improved upon. Only recently have clinics become mainly face to face again, yet in many cases there is little need. A systematic review found equal efficacy between face-to-face and remote consultations in primary care and mental health clinics, albeit with higher discontinuation rates among the teleconsultation groups.^11^ A physician can make a significant impact on air pollution and emissions by working with patients to determine which stages of their clinical journey could be held remotely. These may be equally as clinically effective when employed appropriately and future investments should aim to streamline and improve upon existing remote clinics.

Moving forwards, low carbon medical investigations and treatments are a key element to reaching net-zero. During my own academic work I have seen how rigorous grant applications are, including thorough costing and future applicability, but in my own experience there was insufficient weight placed on the environmental impact. Senior clinicians creating local guidelines should consider the carbon impact of their first line managements as well as opting for prevention and lean pathways where possible, while senior academic physicians should campaign for funding bodies to include sustainability as a mandatory component in grant applications. Without future research in new low-carbon medicines, there is a natural limit to the progress we can make; there is an ethical responsibility to physician researchers to consider the environmental impact of their future work.

Grass-roots change by individuals can be as effective as systemic changes. Fossil fuels are a key culprit in the climate emergency, given their role in creating air pollution and leading to increasing temperatures and thus altered rainfall patterns. One of the biggest individual changes that we can make is opting for preventative healthcare over medical management; this is an area of primary care that I felt was most rewarding, given the potential impact it held for patients. The transportation of medicines is a significant contributor to the emissions within healthcare; the production and transportation of healthcare-related goods accounts for over 80% of healthcare emissions.^12^ We have daily opportunities to limit our prescribing while simultaneously educating and empowering our patients to make healthier choices that also benefit the environment. Cardiovascular disease is an increasing burden on the UK population and every doctor will have daily opportunities to empower patients to implement lifestyle changes that additionally benefit the environment. Reducing fatty red meat intake can drastically improve cardiovascular health and reduce the risk of type 2 diabetes and colorectal cancer, while significantly reducing one’s carbon footprint.^13^ Additionally, encouraging patients to walk all or some of their journey instead of driving will reduce emissions and improve cardiovascular health and obesity. This will ultimately reduce the need for medications arising from cardiovascular and metabolic complications such as statins, anti-hypertensives and diabetic medications. Many of my patients will spontaneously discuss a desire to be more active when they notice my own cycling gear in the consultation room, reminding me of how physicians are often seen as role models. However, many of the patients I speak to have not been properly informed on the influence that these small changes can have on their health and the environment.

While prevention will have the highest impact on emissions, high-quality, cost-effective and green prescribing practices are an effective tool. In particular, de-prescribing can improve both patient compliance and reduce waste. It is a skill that I am in the process of trying to make into a habit of my practice and has been uniformly well received by patients. Additionally, prescribing low-carbon options can minimise medication-related emission, such as opting for tablets over liquid medications or dry powders over propellant inhalers^12^ Green social prescribing is an under-utilised tool in the battle against the climate emergency and can be particularly beneficial for the growing number of patients experiencing loneliness or mental illness. There are a growing number of green charities that patients can be signposted to, for example community gardening projects with the Royal Horticultural Society or conservation work with The Conservation Volunteers. These activities can have a cathartic effect on a patient’s psyche with minimal cost to the NHS, while also helping the planet.

Separate to medication, the waste created by the NHS carries a significant carbon footprint. In the wake of the COVID-19 pandemic, the use of personal protective equipment has stayed high, leading to unnecessary plastic waste, while also being shown to reduce hand hygiene measures due to false reassurance from wearing gloves.^14^ Success has already been seen in the Royal College of Nurses ‘gloves off’ campaign; doctors can make a choice regarding the single-use items they use each day. This is a simple change that physicians can make to reduce the carbon footprint of the workplace, and utilising more low-carbon options on a day-to-day basis is a key step in tackling the climate crisis. However, in addition, systemic investment within the NHS in lowcarbon products will be necessary to create lasting change.

## Conclusion

The scale of the climate crisis is daunting and the idea that a doctor cycling to work will save the world might be laughable. However, doctors’ influence runs deep into their community, and their words and actions can change the lifestyles of many. Physicians can, and should, use their influence to enact systemic change via activism and campaigning, while continuing to implement sustainability changes in their day-to-day practices. The impact of research on the climate crisis should be a key component of future grants. It is in every doctor’s interests to make and promote these changes to protect their patients’ health and limit the damage being done to the planet.

## CRediT authorship contribution statement

**Thomas Hughes:** Writing – review & editing, Writing – original draft, Conceptualization.

## Declaration of competing interest

The author declares that they have no known competing financial interests or personal relationships that could have appeared to influence the work reported in this paper.
